# Optimizing Spray-Dried Porous Particles for High Dose Delivery with a Portable Dry Powder Inhaler

**DOI:** 10.3390/pharmaceutics13091528

**Published:** 2021-09-21

**Authors:** Yoen-Ju Son, Danforth P. Miller, Jeffry G. Weers

**Affiliations:** 1 Genentech, South San Francisco, CA 94080, USA; son.yoen-ju@gene.com; 2 Cystetic Medicines, Inc., Burlingame, CA 94010, USA; dmiller@cysteticmedicines.com

**Keywords:** packing density, product density, small porous particles, corrugated particles

## Abstract

This manuscript critically reviews the design and delivery of spray-dried particles for the achievement of high total lung doses (TLD) with a portable dry powder inhaler. We introduce a new metric termed the product density, which is simply the TLD of a drug divided by the volume of the receptacle it is contained within. The product density is given by the product of three terms: the packing density (the mass of powder divided by the volume of the receptacle), the drug loading (the mass of drug divided by the mass of powder), and the aerosol performance (the TLD divided by the mass of drug). This manuscript discusses strategies for maximizing each of these terms. Spray drying at low drying rates with small amounts of a shell-forming excipient (low Peclet number) leads to the formation of higher density particles with high packing densities. This enables ultrahigh TLD (>100 mg of drug) to be achieved from a single receptacle. The emptying of powder from capsules is directly proportional to the mass of powder in the receptacle, requiring an inhaled volume of about 1 L for fill masses between 40 and 50 mg and up to 3.2 L for a fill mass of 150 mg.

## 1. Introduction

Traditionally, large doses of inhaled drugs have been administered by jet nebulization. High dose nebulized drugs include Virazole^®^ (ribavirin inhalation solution) for the treatment of respiratory syncytial virus [1,2], Nebupent^®^ (pentamidine inhalation solution) for prophylaxis against *Pneumocystis jiroveci* in immunocompromised AIDS/HIV and organ transplant patients [3,4], and TOBI^®^ (tobramycin inhalation solution) for the treatment of *Pseudomonas aeruginosa* infections in cystic fibrosis (CF) patients [5,6].

Jet nebulizers have limitations that can impact patient adherence [7]. They are bulky, noisy, require a power source, and have a high daily treatment burden. The daily treatment burden considers not only the time to administer the drug but also the time for set-up, disassembly, cleaning, and disinfection of the delivery device. Compliance with cleaning nebulizers is typically poor [8] and this can lead to contamination of the nebulizer with bacteria, possibly increasing the risk of administration of new, more virulent pathogens to at-risk patients during treatment [9]. Aqueous solutions of drugs for inhalation often must be reconstituted from lyophilized powder or stored at refrigerated temperatures to maintain chemical stability of the drug substance. Jet nebulizers also produce high levels of fugitive aerosol.

The higher delivery efficiency of vibrating mesh nebulizers can decrease the daily treatment burden [10,11] but this often requires nebulization of hypertonic solutions that pose greater risk of causing irritation in the lungs [12].

Relative to jet nebulizers, dry powder inhalers enable dramatic decreases in administration time and daily treatment burden. The transition from a nebulized treatment with tobramycin inhalation solution (TOBI^®^) to tobramycin inhalation powder (TOBI^®^ Podhaler™) led to a reduction in administration time by ~30 min/day [13,14]. This translated into a high preference for the inhaled powder among CF patients [13] with improvements in adherence [15,16] and pharmacoeconomics [16,17]. Portable dry powder inhalers do not require a power source and are easy to carry in a pocket or purse, enabling discreet use outside the home [14].

### 1.1. High Dose Delivery with Portable Dry Powder Inhalers

**Figure 1:**
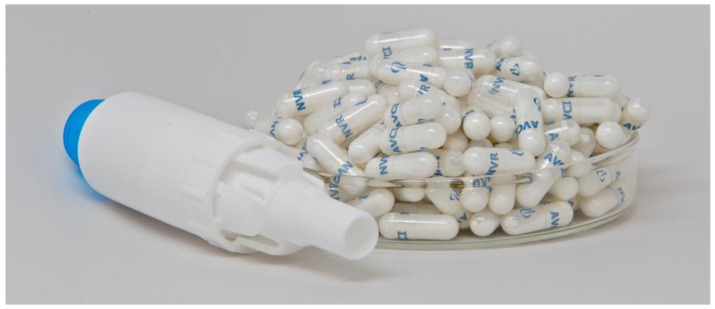
The unit dose Podhaler DPI and a month’s supply of 240 capsules.

Most subjects can empty 40–50 mg of powder from a receptacle in a single inhalation [Section 4.3 and Section 4.4]. As the dose increases and powder mass exceeds 100 mg, the options become less satisfactory. This is exemplified by TOBI Podhaler (powder mass = 194 mg) and Bronchitol^®^ (powder mass = 400 mg), which require administration of four and ten capsules twice daily, respectively (Figure 1). There remains a clear unmet need for improving drug delivery with a portable inhaler when the inhaled powder mass exceeds 100 mg.

Indeed, Hickey et al. defined the challenges associated with high dose delivery well: “Effective high dose delivery of inhaled dry powders is a balance of the influence of product performance (drug formulation, metering, and device) and dose delivery (mass on a single breath, number of breaths per dose) with respect to patient adherence to therapy over potentially long periods of treatment.” [18].

### 1.2. Definition of Ranges of Total Lung Dose

Sibum et al. [19] proposed a definition of high powder doses based on the highest mass of drug that can be delivered with standard adhesive mixtures comprising micronized drug and coarse lactose carrier particles (i.e., lactose blends). They suggested that the upper bound for drug loading in lactose blends is 0.1 mg/mg, after which the drug may not be associated with the carrier and the blend may be mechanically unstable with poor dose reproducibility [20]. The maximum fill mass for marketed adhesive mixtures is 25 mg, resulting in a breakpoint at nominal doses ≥2.5 mg.

**Figure 2:**
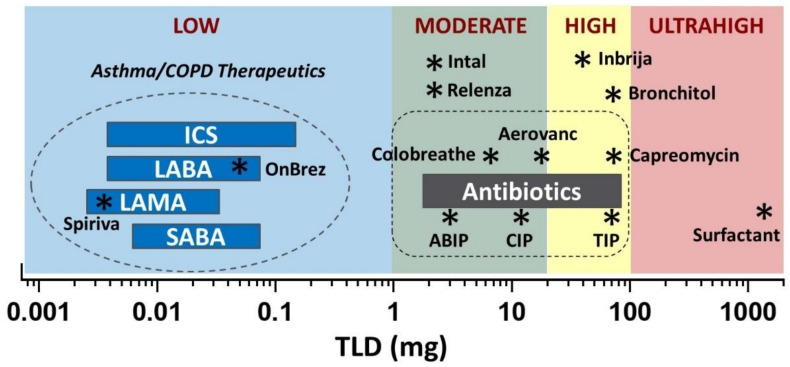
The total lung dose, TLD  (*) of various inhaled drugs. The TLD is divided into low, moderate, high, and ultrahigh dose categories. Abbreviations: ICS, inhaled corticosteroid; LABA, long-acting beta-agonist; LAMA, long-acting muscarinic antagonist; SABA, short-acting beta-agonist; ABIP, amphotericin B inhalation powder (Nektar Therapeutics, San Francisco, CA, USA); CIP, Ciprofloxacin DPI (Bayer, Leverkusen, Germany); TIP, TOBI Podhaler (Novartis, Basel, Switzerland).

Given that inhalation products have markedly different aerosol performance metrics, we prefer to use the total lung dose (TLD) as the defining metric. Adhesive mixtures comprising a force control agent such as magnesium stearate can achieve a TLD of about 0.5 mg/mg of the nominal dose [21,22]. Hence, the breakpoint between low and high doses would equate to a TLD of ~1 mg. Potent asthma and COPD therapeutics (e.g., inhaled corticosteroids and bronchodilators) have TLD values less than ~0.1 mg, falling well within the low-dose classification (Figure 2). Figure 2 also delineates less potent drugs subdivided into three additional groups: moderate, high, and ultrahigh total lung doses. To put the delivery challenge in perspective, this dose range covers six orders of magnitude.

As discussed, there is a limit to the mass of powder that can be inhaled in a single inhalation using a capsule-based dry powder inhaler. Porous particle formulations have achieved a TLD up to ~20 mg in a single inhalation [23]. We define the range of TLD from 1 to 20 mg as moderate doses. For inhaled antibiotics, this group includes Ciprofloxacin DPI, Amphotericin B Inhalation Powder (ABIP), Aerovanc^®^, and Colobreathe^®^. The TLD range from 20 to 100 mg may require either multiple receptacles and/or multiple inhalations from a large receptacle to deliver the TLD. This dose range is defined as high doses and includes products such as TOBI Podhaler (TLD ~70 mg), Bronchitol^®^ (TLD ~70 mg), and Inbrija^®^ (TLD ~40 mg). Finally, TLD values above 100 mg are defined as ultrahigh doses. This group currently has no approved products but includes aspirational products such as pulmonary surfactant (TLD > 1000 mg, assuming a 20 mg/kg dose to a 60 kg adult).

At some point, the TLD can become impractical for delivery with a portable DPI, as it would require an excessive number of receptacles and too many inhalations per receptacle. This practical limit is likely less than 1 g unless a significant innovation in drug delivery is achieved. It is possible that these high doses could be delivered with a powder nebulizer over multiple breaths, but this may encompass many of the challenges associated with liquid nebulizers [24].

The balance of this review is focused on how to maximize the TLD that can be delivered from a given sized receptacle for spray-dried powders. To aid in this endeavor, we introduce a new metric termed the ‘product density’.

## 2. Product Density

The product density ($\rho_{product}$) is simply the TLD achieved with a portable DPI divided by the volume of the receptable ($V_{r}$) that contains the powder. Both terms can be easily measured experimentally. The two most common types of receptacles in non-reservoir-based DPIs are capsules and laminated foil-foil blisters. The receptacle volume of blisters is typically between 0.03 and 0.2 mL, while capsule volumes vary between about 0.2 and 1.0 mL. Some recent DPI designs contain larger volume blisters and use compressed ‘pucks’ to enable larger fill masses [25,26,27]. The TLD can be determined in vivo by imaging (e.g., gamma scintigraphy) [28] and pharmacokinetic methods [29]. Alternatively, the TLD can be estimated in vitro with anatomical throat models (e.g., an Alberta idealized throat, AIT) [30,31]. Estimates can also be made using cascade impactor data but given the dependence of regional deposition on both size and flow rate, it is more appropriate to use a stage grouping (e.g., the stage grouping from stage 3 to the micro-orifice collector (MOC) in a Next Generation Impactor) as opposed to a cutoff size [32,33]. This stage grouping corresponds to particles with an impaction parameter less than ~467 μm^2^ L min^−1^ [32]. Unfortunately, the ‘type’ of aerosol performance data available varies from product to product. When available, priority is given to the use of in vivo imaging and pharmacokinetic results, followed by in vitro anatomical throat data, and then in vitro impactor data. While there may be differences in the in vivo and in vitro measures of TLD, we do not believe that these differences materially impact the results and conclusions of the study.

The product density can be subdivided into three terms that capture the essence of key design features of a high dose product (Equation (1)).

(1) $$\rho_{\mathrm{product}}=\frac{\mathrm{TLD}}{V_{r}}=\left(\frac{m_{\mathrm{powder}}}{V_{r}}\right)\;\left(\frac{m_{\mathrm{drug}}}{m_{\mathrm{powder}}}\right)\;\left(\frac{\mathrm{TLD}}{m_{\mathrm{drug}}}\right)\;$$

The three terms comprise: (1) the mass of powder (*m*_powder_) that can be filled into the receptacle volume (*‘packing density’*), (2) the mass of drug (*m*_drug_) divided by the mass of powder (*‘drug loading’*), and (3) the fraction of the drug that is delivered into the lungs (*‘aerosol performance’*).

### 2.1. Packing Density

The packing density depends on the characteristics of the powder and the specifics of the filling process. The density of the powder bed depends not only on the particle density but also on how close the particles are able to be packed in the powder bed, which depends on the cohesive forces between particles.

Fine, cohesive powders are typically filled with vacuum drum fillers. These fillers, originally designed during the development of Exubera^®^, utilize ultrasonic energy to induce powder flow from a trough into a truncated cone-shaped filling cavity located on a rotating cylinder [34,35,36]. Acoustic energy may also be used to fluidize powder in the trough [36]. Drum fillers enable accurate and precise filling of fine cohesive powders down to fill masses of ~1 mg with an RSD of ≤2% [34]. The loosely compacted pucks are easily broken up during a ‘capsule polishing’ step where the surface of the capsules is dedusted.

The size of the void space within the receptacle depends on the size and shape of the receptacle and the corresponding size and shape of the puck as well as the number of pucks filled. The volume of the receptacle filled may also be influenced by the free volume needed within the receptacle for effective powder emptying. For example, for blister-based receptacles, powder properties (e.g., physical stability and chemical stability) can be negatively impacted if the powder is touching the lid foil during heat sealing.

### 2.2. Drug Loading

In the term drug loading, the ‘drug’ comprises only the active drug substance. It does not include the mass of the counterion in salts (or conformer in cocrystals), the mass in a drug that is not part of the active agent (e.g., in a prodrug), or the mass associated with excipients and residual process aids. For high dose delivery, it is highly desirable to maximize the drug loading in the formulation. With that said, the use of excipients is often necessary and, as we will show, critical for optimizing high dose delivery (Section 4).

### 2.3. Aerosol Performance

Aerosol performance depends on the nature of the formulation and the delivery device, and how the two components work in concert to optimize drug delivery to the target site within the lungs.

### 2.4. Optimization of Product Density

Taken together, the three terms encompass all aspects of the formulation, manufacturing process, and product performance, hence the term ‘product density’. Attempts to improve one term may negatively impact another. For example, in some formulations, increasing powder compression during filling to increase the packing density may diminish aerosol performance. Drug delivery with neat formulations to increase drug loading may also reduce aerosol performance or increase dosing variability. Indeed, the constraints imposed for high dose delivery are unique for each drug being considered and the development of the drug product often becomes an optimization exercise. There is no single right answer and there are typically multiple approaches to arrive at an acceptable drug product, especially for drugs that require a moderate TLD.

## 3. Product Densities in Inhaled Drug Products

The product density metrics for the drug products pictured in Figure 2 are presented in Table 1.

### 3.1. Low Dose Products

As discussed, drugs for asthma and COPD are highly potent with TLDs less than ~0.1 mg (Figure 2). Table 1 includes product density metrics for two COPD drug products comprising adhesive mixtures: Spiriva^®^ HandiHaler^®^ (Boehringer Ingelheim, Ingelheim am Rhein, Germany) [37,38] and Onbrez^®^ Breezhaler^®^ (Novartis) [22,39]. The drugs in these products are bronchodilators: tiotropium bromide, a muscarinic receptor antagonist, and indacaterol maleate, a beta-agonist. Owing to the low nominal doses required, the drug loadings in these formulations are very low (0.003 and 0.006 mg/mg, respectively). The fill masses and resulting packing densities are also low (18.3 and 83.3 mg/mL, respectively), as are the TLD values (0.20 and 0.34 mg/mg, respectively). These metrics result in product densities for Spiriva and Onbrez of 0.012 and 0.17 mg/mL, respectively.

The magnitude of the flow rate dependence for these formulations was assessed using the $Q_{index}$ [40]. The $Q_{index}$ is the percent difference in TLD between pressure drops of 1 and 6 kPa, normalized by the higher of the two values. Formulations with $Q_{index}$ values <15% are defined as having low flow rate dependence, between 15 and 45% medium flow rate dependence, and >45% high flow rate dependence [40]. The $Q_{index}$ values for Spiriva HandiHaler and Onbrez Breezhaler were −25.0 and −31.0%, respectively, indicative of medium flow rate dependence [40]. A medium flow rate dependence is characteristic of lactose blends [40].

### 3.2. Intal^®^ (Cromolyn Sodium Inhalation Powder)

Cromolyn sodium (also known as sodium cromoglycate) is a bronchodilator developed from the plant extract Khellin. At the time it was developed, Intal (Fisons, Loughborough, UK) was considered to be a high dose product, delivering 20 mg of micronized cromolyn sodium to asthma patients from a size 2 capsule with the Spinhaler^®^ DPI (packing density = 54.1 mg/mL). The drug loading is quite high (0.91 mg/mg) owing to formulation as the disodium salt (i.e., without excipients). The TLD varies between 0.055 and 0.17 mg/mg with variations in inspiratory flow rate (mean = 0.113 mg/mg) [41], leading to a product density of ~5.4 mg/mL. On average, the TLD/capsule is about 2 mg. The $Q_{index}$ for Intal is +73.7%, indicative of a high flow rate dependence [40].

### 3.3. Relenza^®^ (Zanamivir Inhalation Powder)

Relenza (GSK) is a dry powder formulation of the antiviral zanamivir, used for the treatment of influenza. The drug product comprises an adhesive mixture of 5 mg of micronized drug blended with 20 mg of coarse lactose carrier particles (drug loading: 0.2 mg/mg) [42]. Twenty-five milligrams of the formulated powder was filled into laminated foil blisters (*V_r_* ~ 0.16 mL) for a packing density of 125.0 mg/mL. A dose consists of administration of the contents of two blisters (total powder dose = 50 mg). The TLD of 0.23 mg/mg was determined with an anatomical throat model [43], resulting in a product density of 5.8 mg/mL. The TLD/blister is ~1.2 mg or ~2.4 mg/dose.

### 3.4. Colobreathe^®^ (Colistimethate Powder for Inhalation)

Colobreathe (Forest Laboratories) is approved in Europe for the treatment of chronic *Pseudomonas aeruginosa* infections in cystic fibrosis (CF) patients [44,45]. Colobreathe delivers an emitted dose of 125 mg of neat colistimethate sodium from the Turbospin^®^ dry powder inhaler twice daily [44,45]. The packing density is high (391.9 mg/mL) as the entire 145 mg powder dose is loaded into a single size 2 hard gelatin capsule. Colistimethate sodium is a prodrug of colistin that is intended to improve the biocompatibility of the cyclic peptide [46]. Colistin is first derivatized with methanesulfonic acid, and then converted into the sodium salt [46]. This results in a drug loading of the active colistin drug substance of ~0.44 mg/mg [46]. The aerosol performance of Colobreathe is poor compared to the spray-dried formulations, detailed below, with a total lung dose determined by gamma scintigraphy of 0.12 mg/mg [47]. These factors contribute to a product density of just 20.7 mg/mL. If colistimethate is treated as a neat drug (i.e., not as a prodrug), the product density increases to 46.1 mg/mL, comparable to the small porous particles detailed below. The in vitro TLD of neat colistimethate powder measured in the idealized Alberta throat model with CF patient inspiratory flow profiles, is highly dependent on flow rate ($Q_{index}$ = −45.8%). The low TLD is also expected to lead to significant variability in TLD associated with differences in the anatomical features of the oropharynx among patients [48,49].

### 3.5. TOBI^®^ Podhaler™ (Tobramycin Inhalation Powder)

TOBI Podhaler (Novartis) is also administered to CF patients as a maintenance treatment for chronic *P. aeruginosa* infections in the airways [13,14,16]. The drug product was developed as a life cycle extension to the nebulized TOBI^®^ drug product, significantly decreasing the burden of treatment while also improving patient convenience [13,14,16]. TOBI Podhaler is prepared using the solution-based PulmoSphere™ manufacturing process [14,50] wherein tobramycin sulfate is dissolved in the continuous phase of a perfluorooctyl bromide-in-water emulsion [14,50]. During drying, the evaporating liquid in the emulsion droplets leaves behind nanopores in the dried particles.

On a dry basis, the spray-dried drug product comprises 85% tobramycin sulfate and 15% excipients (2:1 molar ratio of distearoylphosphatidylcholine (DSPC):calcium chloride) [51]. When expressed in terms of tobramycin content and including residual solvents, the drug loading is 0.58 mg/mg with ~20% of the mass in the composition taken up by the sulfate counterion. The small porous particles have a median geometric size of about 3 μm. The drug product is filled into a size 2 HPMC capsule with a 48.5 mg fill mass yielding a packing density of 131.1 mg/mL. The nominal dose of 112 mg (28 mg per capsule) is administered in four capsules with the Podhaler dry powder inhaler twice daily, for a daily dose of 224 mg/day. The emitted dose is greater than 90% with about 63% of the nominal dose delivered into the lungs [52]. This results in a product density of 47.1 mg/mL and a TLD/capsule of 17.6 mg. The TLD in TOBI Podhaler is independent of inspiratory flow rate over the range of breathing profiles typically achieved by CF patients ($Q_{index}$ = +0.3%) [40,52].

### 3.6. Ciprofloxacin DPI (Ciprofloxacin Powder for Inhalation)

Ciprofloxacin is a broad-spectrum antibiotic with activity against both Gram-positive and Gram-negative bacteria. Ciprofloxacin DPI (Bayer) was developed for the treatment of chronic infections in CF and non-CF bronchiectasis patients [53,54]. Ciprofloxacin DPI is prepared by the suspension based PulmoSphere manufacturing process, using the poorly soluble zwitterionic form of the drug at neutral pH [50,54]. This process results in small porous particles of micronized crystalline drug particles coated with porous shell comprising a 2:1 molar ratio of DSPC:CaCl_2_. Formulation of the neutral form of the drug provides for dissolution-limited delivery in the lungs, enhancing the pharmacokinetic/pharmacodynamic metrics (e.g., the AUC/MIC ratio) and efficacy of the drug in the lungs [54]. The drug loading in the spray-dried drug product is 0.65 mg/mg. Using a drum filler, 50 mg of the powder is loaded into size 2 HPMC capsules, giving a packing density of 135.1 mg/mL. The nominal dose of the drug product is 32.5 mg, with delivery from a single capsule twice daily (65 mg daily dose). The aerosol performance as determined by gamma scintigraphy is about 0.53 mg/mg [55]. These results provide a product density of 46.5 mg/mL and a TLD/capsule of 17.2 mg. Moreover, as per TOBI Podhaler, the $Q_{index}$ is low ($Q_{index}=$−12.2%) [54].

### 3.7. Amphotericin B Inhalation Powder (ABIP)

Amphotericin B inhalation powder (Nektar Therapeutics) was developed for prophylaxis of immunocompromised patients against the development of invasive fungal infections [56,57,58]. The drug product completed end-of-Phase 2 meetings with health authorities, but the program was discontinued after the pulmonary business unit of Nektar was sold to Novartis. The suspension based PulmoSphere formulation comprised 50% *w*/*w* crystalline amphotericin B with the remainder being the PulmoSphere excipients (i.e., a 2:1 mol:mol ratio of DSPC:CaCl_2_) [56]. The drug is administered with a loading dose followed by weekly maintenance doses [58]. Given the relatively low maintenance dose (5 or 10 mg), the fill mass occupies only a small percentage of the size 2 capsule volume. As a result, the packing density is just 27.0 mg/mL. The aerosol performance of ABIP is independent of flow rate and inhaled volume with an emitted dose of 96 ± 3%, an MMAD of 2.3 μm, an FPF_S3-F_ of 83% of the nominal dose, and a $Q_{index}$ of just +1.3% [56]. The TLD is about 0.7 mg/mg. This results in a product density of about 9.5 mg/mL, and a TLD/capsule of about 3.5 mg.

### 3.8. Capreomycin Inhalation Powder

Capreomycin is an antibiotic that is commonly used in the treatment of tuberculosis in combination with other antibiotics. Core-shell particles of capreomycin sulfate (~87% capreomycin) were prepared by spray drying the drug with leucine as a shell-forming excipient [59,60]. On a dry basis, the drug product contained 16.7% *w*/*w* leucine. The spray-dried powder had a water content of 5.3% *w*/*w*, resulting in a drug loading of ~0.69 mg/mg. Thirty milligrams of powder was filled into size 3 capsules (packing density = 100.0 mg/mL) and the contents of up to 12 capsules (total powder dose of 360 mg) were administered to healthy adult subjects [60]. The MMAD of capreomycin inhalation powder was 4.74 μm. The FPF_S2-F_ was 50% of the nominal dose and the FPF_S4-F_ was 10% of the nominal dose. Splitting the difference yields an FPF_S3-F_ of about 30% of the nominal dose. Overall, this results in a packing density of 20.7 mg/mL and a TLD/capsule of 6.2 mg. The TLD for the full 12 capsules was about 74.5 mg, i.e., comparable to that of the TOBI Podhaler.

### 3.9. Aerovanc^®^ (Vancomycin Inhalation Powder)

Aerovanc (Savara Pharmaceuticals) is intended to treat Gram-positive infections (e.g., methicillin-resistant *Staphylococcus aureus,* MRSA) in CF patients. The small porous particles were manufactured by spray drying a solution-based liquid feed that contained 83% vancomycin and 17% excipients [61,62,63]. The core-shell particles contained vancomycin in the core of the particle with a shell of leucine. Thirty milligrams of Aerovanc was filled into size 3 HPMC capsules providing a packing density of 100.0 mg/mL [61]. Aerosol delivery into the lungs and systemic circulation was about 0.50 mg/mg [61,62]. Overall, these features resulted in a product density comparable to the other small porous particle formulations, i.e., 41.5 mg/mL and a TLD/capsule of 12.5 mg.

### 3.10. Bronchitol^®^ (Mannitol Inhalation Powder)

Bronchitol (mannitol inhalation powder) improves mucociliary clearance in CF patients [64,65,66]. The 400 mg nominal dose of mannitol is subdivided into ten size 3 capsules (V_r_ = 0.30 mL), each containing 40 mg of the powder formulation (packing density = 133.3 mg/mL). The drug product is administered twice daily for an 800 mg daily nominal dose. Bronchitol is manufactured by spray drying a solution of neat mannitol (drug loading = 1.0 mg/mg) to produce fine crystalline drug particles. The aerosol performance of spray-dried mannitol with a variant of the RS01 dry powder inhaler (Osmohaler™, *R* = 0.036 kPa^0.5^ L^−1^ min) was determined by SPECT imaging in nine healthy subjects [66]. The measured TLD was 0.175 mg/mg [66]. This leads to a product density of 23.3 mg/mL and a TLD/capsule of 7.0 mg. The TLD per dose is ~70 mg. The treatment burden is high with Bronchitol with the need for patients to load and inhale the contents of twenty capsules daily.

As with the other neat drug formulations (i.e., Intal and Colobreathe), the flow rate dependence is high. The flow rate dependence of inhaled mannitol was assessed with the lower resistance version of the Osmohaler (*R* = 0.021 kPa^0.5^ L^−1^ min) used for bronchoprovocation testing. The TLD decreased from 0.199 mg/mg at a flow rate of 67 L min^−1^ to 0.098 mg/mg at a mean flow rate of 121 L min^−1^, corresponding to a high $Q_{index}$ of −52.0% [66].

### 3.11. Inbrija^®^ (Levodopa Inhalation Powder)

Inbrija (Acorda Therapeutics) is prescribed to Parkinson’s patients during their “off-period” when symptoms are high and plasma dopamine levels are low [67]. Pulmonary administration enables rapid increases in plasma dopamine. The drug product on a dry basis comprises 90% *w*/*w* levodopa with the remaining 10% *w*/*w* being a mixture of dipalmitoylphosphatidylcholine (DPPC) and sodium chloride [25,68,69,70]. The spray-dried drug product also contains residual moisture. Inbrija is manufactured as large porous particles (ARCUS™ technology) as first described by Edwards et al. [71]. The median geometric diameter of the corrugated particles is about 6 to 8 μm [70]. The large size necessitates that the particle density be low to make the particles respirable. Indeed, the poured bulk density of the large porous particles is about 0.02 to 0.05 g/cm^3^ [70]. Hence, the volume of powder that must be inhaled is large. About 50 mg of powder containing 42 mg of levodopa is filled into size 00 HPMC capsules (*V_r_* = 0.95 cm^3^), resulting in a packing density of about 52.6 mg/mL and a drug loading of 0.84 mg/mg. Despite the large volume of powder, emptying of the capsule typically requires only a single inhalation by the patient.

Patients with Parkinson’s disease take the drug on demand up to five times daily for a maximum daily nominal dose of 420 mg (two capsules per dose) [25]. The drug is administered with the Inbrija dry powder inhaler, a capsule-based inhaler derived from the Turbospin aerosol engine and adapted to use a size 00 capsule. The in vitro emitted dose is about 86% with approximately 50% of the drug delivered into the lungs (i.e., aerosol performance ~ 0.5 mg/mg) [23,70]. This leads to a product density of about 22.1 mg/mL and a TLD/capsule of 21.0 mg.

## 4. Increasing TLD and Product Density in Spray Dried Powders

The discussion that follows is not intended to be an exhaustive review of manuscripts related to high dose delivery of inhaled therapeutics.

Here, we use specific examples to illustrate trends in the field and their impact on the TLD and product density. For a more comprehensive review of the literature, readers should consult the recent themed issue edited by Das, Stewart, and Tucker [72], which contains numerous reviews of interest [19,73,74,75,76,77,78,79], as well as other recent reviews dedicated to high dose delivery [80,81,82].

### 4.1. Why Not Just Formulate Neat Drug?

As suggested, there is currently a limit to the mass of powder that can be administered in a single inhalation. Hence, there is a strong desire to maximize the $TLD/m_{powder}$. This ratio is simply the product of the last two terms in the product density (Equation (1)). Stated another way, there is a desire to increase the drug loading (i.e., minimize or eliminate excipients including counterions and coformers) and to maximize aerosol performance in these high dose formulations. One group has gone so far as to suggest that the improvements in aerosol performance observed with spray-dried particles in Table 1 (TLD~40–70% of the nominal dose) can be duplicated through improvements in device design and that “designing and developing more powerful DPIs seems a better solution for improving dispersion” [19]. It remains to be seen whether this can be realized with more cohesive powders sans excipients and whether this will compromise the consistency of dose delivery (e.g., via increases in flow rate dependence and variability associated with oropharyngeal filtering of particles, as was observed with the neat Intal, Bronchitol, and Colobreathe formulations) [40,66]. The product density metrics for these examples are presented in Table 2.

It is worth noting that formulating neat crystalline drugs with a high true density does not necessarily translate into a high packing density or tapped density [96]. Indeed, the tapped densities achieved with neat crystalline drug powders, especially particles of respirable size, are often similar to what is observed with spray-dried particles. This is due to a low packing density resulting from significant porosity in the powder bed.

#### 4.1.1. Neat Jet-Milled Clofazimine

Clofazimine is used in combination with rifampicin and dapsone in the treatment of leprosy. There is growing evidence that the drug may also have activity in the treatment of non-tuberculosis mycobacterial infections [97].

Brunaugh et al. [83] assessed the aerosol performance of neat jet-milled clofazimine powder (drug loading: 1.0 mg/mg) administered with the low resistance RS01 DPI (Table 2). The milling process resulted in a median particle diameter by laser diffraction of 1.8 μm. Twenty milligrams of the powder was loaded into a size 3 capsule for a packing density of 66.7 mg/mL. The aerodynamic particle size distributions were determined on a Next Generation Impactor at pressure drops of 1 and 4 kPa, corresponding to flow rates of 47 and 93 L min^−1^, respectively. The fine particle dose on stages 3 to MOC (i.e., FPD_S3-F_) were approximately 9.5 and 8.5 mg, respectively, suggesting that throat deposition increases at the very high flow rates achieved with the low resistance device. The $Q_{index}$ shows a medium flow rate dependence with a value of −17.6%. For the purposes of the product density calculation, the mean of the two FPD_S3-F_ values was used, i.e., 9.0 mg. This corresponds to an estimated TLD of 0.45 mg/mg. Overall, the product density was ~30.0 mg/mL, or about 50% lower than the small porous particle formulations in Table 1.

The TLD/capsule of ~9.0 mg sits comfortably in the moderate dose region where many technology solutions exist. The advantage of jet milling is that the process is simple and product development is also simplified by the absence of excipient. The process depends critically on the ability to effectively mill the drug to respirable sizes and the interparticle cohesive forces in the resulting agglomerated drug particles. As such, this process is not universal for all drugs. The use of shell-forming excipients was introduced, in part, to control the surface properties independent of the nature of the drug substance.

#### 4.1.2. Tobramycin Base Formulation—1

A number of studies have been conducted with inhaled tobramycin. These studies are motivated by the desire to reduce the daily treatment burden by increasing the product density. As discussed, approximately 20% of the mass in the TOBI Podhaler spray-dried particles is from the sulfuric acid used to make the sulfate salt. As such, formulation as the free base has the potential to significantly increase the drug loading in tobramycin formulations.

Pilcer et al. [84] utilized gamma scintigraphy to determine the TLD for two high drug loading tobramycin free base formulations in nine patients with CF. Tobra Form 1 comprised lipid-coated crystals of tobramycin manufactured by spray drying a suspension of tobramycin base in isopropanol with 5% dissolved lipids (3:1 w:w ratio of cholesterol to Phospholipon). Tobra Form 2 was simply neat micronized tobramycin free base. Twenty-five milligrams of each powder was filled into size 3 capsules (packing density = 83.3 mg/mL). The capsules were loaded into an RS00 DPI. The TLDs for Tobra Form 1 and Tobra Form 2 were 0.53 and 0.34 mg/mg, respectively. The interpatient variability in the TLD increased from 19% for Tobra Form 1 to 36% for Tobra Form 2, consistent with previous studies where interpatient variability in the TLD decreased with increases in TLD [48,49].

This example illustrates the potential for suspension-based spray drying to deliver lipid-coated crystals with good aerosol performance with use of just 5% *w*/*w* lipid as a shell former. It also further illustrates the challenges associated with neat drug formulations, both from aerosol performance and variability in dose delivery perspectives.

#### 4.1.3. Tobramycin Base Formulation—2

Buttini et al. [85,86] combined a solution of tobramycin base in water with an alcoholic solution of sodium stearate to form a 1% *w*/*v* liquid feed. The liquid feed was then spray dried to form amorphous particles of tobramycin base coated with just 1% *w*/*w* sodium stearate (TobraPS). Accounting for residual water in the formulation, the drug loading was 0.91 mg/mg. The dry powder was loaded into different sized capsules ranging from size 3 to size 0. The capsules were loaded into variants of the RS01 DPI modified to actuate and deliver the different sized capsules. The data for the size 0 capsule are presented in Table 2.

One hundred twenty milligrams of TobraPS powder was loaded into a size 0 capsule (packing density = 176.5 mg/mL). The aerosol performance of the lipid-coated particles was comparable or slightly better than TOBI Podhaler. Owing to the use of tobramycin base and increases in packing density, TobraPS achieved a significantly greater product density (104.4 mg/mL) compared to TOBI Podhaler (47.1 mg/mL). This, coupled with the use of a larger sized receptacle, enabled TobraPS to achieve a comparable TLD to TOBI Podhaler (~70 mg) by administration of TobraPS from a single size 0 capsule. As shown in Section 4.3 and Section 4.4, this fill mass can be emptied by most CF patients in two or three inhalations. Overall, this approach decreases the number of capsules from four to one and the total number of inhalations from eight to two or three, a notable improvement.

#### 4.1.4. Tobramycin Base Formulation—3

Hoppentocht et al. [87,88] spray dried neat tobramycin base from water to form an amorphous powder. The residual water content in the drug product was not disclosed. For the purposes of this estimate of product density, the drug loading is assumed to be 0.95 mg/mg. Thirty milligrams of amorphous tobramycin powder was loaded into the Cyclops single-dose cartridge (SDC) in the single-use disposable DPI. Although not specified, it is assumed that the study was run with the standard SDC that has a 0.35 mL volume. This results in a packing density of about 85.7 mg/mL.

Single ascending doses of 30, 60, 120, and 240 mg (requiring 1–8 devices) were administered to eight adult patients with non-CF bronchiectasis (age range from 57 to 73 years). Local tolerability and systemic pharmacokinetics were assessed. The aerosol performance of Tobra Cyclops was estimated from the systemic bioavailability in comparison with the systemic bioavailability results observed for TOBI Podhaler in CF patients in a Phase 1 clinical study [98]. This estimate assumes that the pulmonary bioavailability of tobramycin is ~100% of the TLD. According to early work on the development of an inhaled biopharmaceutical classification system (iBCS), drugs that are not permeability or dissolution limited typically have pulmonary bioavailabilities approaching 100% [99]. Indeed, the high pulmonary bioavailability of inhaled tobramycin was demonstrated in pharmacokinetic studies by Li and Byron [100]. In the Phase 1 study with TOBI Podhaler, a 112 mg dose was found to be equivalent to a 300 mg dose of TOBI. The dose-normalized AUC_0–12h_/mg was 0.041 h mg/L. The corresponding dose-normalized AUC_0–12h_/mg values for Tobra Cyclops were 0.013 (30 mg), 0.017 (60 mg), 0.019 (120 mg), and 0.022 h mg/L (240 mg). For the two higher doses, the relative bioavailability of Tobra Cyclops is about half that observed for TOBI Podhaler. The TLD for TOBI Podhaler was determined in the Alberta idealized throat model (0.63 mg/mg) [52]. This leads to an estimate of the aerosol performance of 0.34 mg/mg and a product density of 27.7 mg/mL for Tobra Cyclops. The TLD/device is ~9.7 mg.

#### 4.1.5. Levodopa Formulation Co-Milled with Force Control Agent

Another manufacturing option is to co-mill the drug substance with a force-control agent (FCA) such as magnesium stearate, leucine, or a phospholipid under high-shear mixing processes such as mechanofusion [101,102].

In this example, Luinstra et al. [89,90] co-milled levodopa with 2% *w*/*w* leucine. Thirty milligrams of the coated drug particles was loaded into the Cyclops single-use disposable DPI (packing density = 85.7 mg/mL) and the systemic pharmacokinetics (i.e., the target for CNS delivery) and tolerability of inhaled levodopa was assessed in eight patients with Parkinson’s disease. The aerosol performance was calculated from the systemic bioavailability of inhaled levodopa from the Cyclops DPI relative to results published for Inbrija and an oral SINEMET 25–100 tablet. SINEMET 25–100 has an oral bioavailability of 71% for levodopa at steady state [23,103,104]. Based on the bioavailability relative to the oral control, Inbrija has a TLD of about 0.46 mg/mg. This is consistent with aerosol performance metrics for Inbrija which suggest a TLD of about 0.50 mg/mg [69,70]. The dose normalized bioavailability of the 30 mg dose of inhaled levodopa from the Cyclops device is about 1.9 times lower than that of Inbrija, suggesting a TLD of about 0.24 mg/mg. This results in a product density of ~20.2 mg/mL and a moderate TLD of ~7 mg/device.

#### 4.1.6. Fixed Dose Combinations

Several groups have explored the development of fixed dose combinations of two or more drugs [95,105,106,107,108]. In many of these studies the more hydrophobic drug is used as a shell-forming material with the more hydrophilic/hygroscopic drug present in the core of the spray dried core-shell particles [95,107]. The shell-forming drug helps to improve the physical stability, chemical stability, and aerosol performance of the drug that is less stable to changes in RH. For antibiotics, the two drugs may also work synergistically to lower the dose needed for effective bacterial killing, thereby decreasing the TLD/mg powder [105,106,108].

For example, Momin et al. [95] studied a fixed dose combination of 60% kanamycin/40% rifampicin spray-dried from a 70/30 % *v*/*v* mixture of ethanol/water with no added excipients (drug loading = 1.0 mg/mg) (designated as FDC R/K RS00 in Table 2). Twenty milligrams of the powder was filled into a size 3 capsule (packing density = 66.7 mg/mL) and the powder was administered with the RS00 DPI at a flow rate of 100 L/min and an inhaled volume of 4 L. Surprisingly, the FPF_S3-F_ differed for the two drugs, with about 0.68 mg/mg of the shell-forming rifampicin and 0.54 mg/mg of the kanamycin deposited on S3-F. The average of the two values was used for the aerosol performance, i.e., 0.61 mg/mg. This resulted in a product density of 40.7 mg/mL and a TLD/capsule of ~12.2 mg (Table 2).

#### 4.1.7. Excipients

Overall, the results presented above suggest that there are options to significantly improve the drug loading in high dose formulations. It is clear that, as the dose increases, a conscious attempt should be made to minimize the percentage of excipients in the formulation. All of the examples presented provide options for moderate TLD delivery. Spray-dried formulations containing small amounts of shell-forming excipients achieve better aerosol performance and improved dose consistency relative to neat drug particles.

While minimizing excipients and process aids is important for high dose formulations, there are many reasons to add excipients beyond improving aerosol performance. Salts are often preferred because of their improved solubility, purity, and crytallizability relative to neutral forms. For labile drug substances, excipients can be used to improve the physical and chemical stability of the drug substance. We add excipients to improve the tolerability and safety of the drug product. We add excipients to improve efficacy by controlling pharmacokinetic/pharmacodynamic metrics. We add excipients to improve the consistency of dosing by reducing variability associated with oropharyngeal filtering of particles, variability associated with inspiratory flow rate, and variability associated with coformulation effects in fixed dose combinations. In addition, yes, we add excipients to reduce interparticle cohesive forces, thereby enabling improvements in aerosol performance, packing density, and targeting within the respiratory tract.

To put this in perspective, let us consider the formulations comprising tobramycin base detailed above. From a regulatory perspective, TOBI Podhaler was filed as a 505(b)(2) NDA, with nebulized TOBI as the reference listed drug (RLD). As such, key features of the drug product (e.g., use of the sulfate salt of the drug substance) were maintained. This allowed the development of TOBI Podhaler to leverage the extensive systemic and pulmonary safety established by TOBI in both nonclinical and clinical studies. This further enabled TOBI Podhaler to go straight from a Phase 1 single ascending dose study in CF patients to Phase 3. The TOBI Podhaler dose for Phase 3 was selected based on its equivalence to TOBI in terms of systemic drug levels [98].

Tobramycin is an aminoglycoside antibiotic containing three glycosidic rings and five primary amine groups. TOBI was formulated at pH 6.0 by the addition of sulfuric acid to tobramycin base to provide a stable formulation. This avoided the use of preservatives and antioxidants (e.g., phenol and sodium bisulfite) which had been demonstrated to contribute to bronchospasm when inhaled by CF patients [6,109]. One challenge with the use of tobramycin base is the high pH of the drug in water (pH~10). The multiple amine groups, high dose, and twice daily administration provides the potential for significant buffering capacity (i.e., raising concerns about altering the physiologic pH in the lungs). The TOBI Inhalation Solution monograph suggests that nebulized liquids can be safely administered below pH 8.7 [6]. While long-term safety studies have been conducted with TOBI and TOBI Podhaler with the sulfate salt, the long-term safety of tobramycin base has yet to be established and, as such, needs to be kept in mind for future life cycle product designs.

The physicochemical stability of the formulated drug product is critical to the development and registration of dry powder inhalation products. This is especially important for amorphous materials which tend to have lower physical and chemical stability. While tobramycin base can be crystallized, salts of tobramycin cannot [51]. As is expected, the stability of the amorphous phase is significantly greater for the salt forms of tobramycin with the sulfate salt having a much higher glass transition temperature than the hydrochloride salt or the free base (at 11.3% RH, the *T_g_* values are 105, 72, and 63 °C, respectively) [51,110]. At 43% RH, the *T_g_* of tobramycin base is equivalent to a storage temperature of 25 °C. Amorphous tobramycin base presents additional physical stability challenges, crystallizing at RH values >53% RH [51,87].

In TOBI Podhaler, the high *T_g_* of tobramycin sulfate and high gel to liquid crystal phase transition temperature of the phospholipid acyl chains in the spray-dried drug product enables room temperature stability (physical, chemical, and aerosol) over a period of three years [14,110]. Maintaining long-term stability requires a delicate balance of physical form, formulation/process, and packaging. The general rule of thumb is that for long-term stability the *T_g_* of the amorphous phase should be at least 50 °C above the storage temperature [111]. For tobramycin base, the low *T_g_* suggests that room temperature stability may be challenging, even when using laminated foil blisters with very low water permeability. This can possibly be mitigated through refrigerated storage with very tight packaging. Peelable blisters such as those used in single-use disposable DPIs may not provide a sufficient moisture barrier to maintain long-term stability of amorphous tobramycin base. Shell formers such as leucine, trileucine, and sodium stearate may be added to reduce the instantaneous drop in aerosol performance when inhalers are operated at high RH [112,113,114,115,116]. They may also slow changes in physical form and drug content on storage.

We raise these points to reinforce the concept that there are many reasons that formulators add excipients (including counterions) to formulations and that these considerations need to be kept in mind during formulation development.

### 4.2. Why Not Take the Air Out?

While significant attention has been paid to maximizing drug loading and aerosol performance in high dose products, comparatively little time has been invested in maximizing the packing density of particles in the receptacle. As discussed, the packing density depends not only on the particle density, but also on the void spaces between particles and details around the filling process.

For porous particles, the aerodynamic diameter, $d_{a},$ is related to the geometric diameter of the particles ($d_{g}$) and the particle density ($\rho_{p}$) by Equation (2):

(2) $$d_{a}=d_{g}\sqrt{\rho_{p}}$$

To achieve an equivalent aerodynamic diameter with larger sized particles, the particle density must be decreased. Given that this relationship is proportional to the square root of particle density, marked reductions in particle density are necessary for modest increases in particle size. This is readily apparent for the spray-dried formulations in Table 1. Small porous particles with $d_{g\;}$= 1–4 μm (e.g., TOBI Podhaler, Ciprofloxacin DPI, Aerovanc) achieve higher packing densities and product densities than large porous particles with $d_{g}\;$= 6–8 μm (e.g., Inbrija) owing, in part, to their greater particle densities (Table 1). The question is whether the particle density can be increased further by reducing particle corrugation or porosity while still maintaining acceptable powder flow and aerosol performance.

#### 4.2.1. Small Dense Particles

Pulmatrix, Inc. developed the iSPERSE™ technology to address this question [92,117]. iSPERSE particles comprise fine spray-dried particles ($d_{g}$ < 5.0 μm) with a tapped density > 0.4 g cm^−3^. The increased tapped density relative to many lower density porous particle formulations is due, in part, to the incorporation of high-density inorganic salts in the particles. Unfortunately, little data have been published demonstrating the capability of the technology.

In one study, Manzanedo et al. [92] studied iSPERSE powders comprising 75–90% levofloxacin with the remainder being a 1:2 w:w mixture of leucine and sodium chloride. For this estimate, we will use the higher drug loading of 0.9 mg/mg. A fill mass of 40 mg in a size 3 capsule was administered with the RS01 DPI (packing density = 133.3 mg/mL). The FPD_S2-F_ was presented and varied between 0.39 and 0.62 mg/mg (mean = 0.51 mg/mg). Obviously, the FPD_S3-F_ will be less than this—let us assume 0.4 mg/mg. This yields an approximate product density of 48.0 mg/mL which is comparable to the values for small porous particle formulations (e.g., TOBI Podhaler, Ciprofloxacin DPI, Aerovanc) listed in Table 1. They claim to be able to fill up to 100 mg in a size 3 capsule (packing density = 333 mg/mL) but provide no data to support this assertion [92].

#### 4.2.2. Coated Crystals

Another path to increasing the particle density is via spray drying of suspension-based liquid feeds comprising crystalline drug particles [53,54,56,84]. In this process, the crystalline particles are typically coated with a layer of shell-forming excipient. Numerous examples of coated crystals are provided in this review, including Ciprofloxacin DPI [53,54], ABIP [56], and Tobra Form 1 [84]. Given that the drug makes up the bulk of the spray-dried particle and most crystalline drugs have true densities >1 g/cm^3^, the particle density of particles prepared from suspension-based liquid feeds is expected to be higher. The fill mass of crystalline Ciprofloxacin DPI is 50 mg (packing density = 135.1 mg/mL). During dose-ranging studies, higher doses of the drug were studied but produced no added clinical benefit [118]. Nonetheless, fill masses up to 75 mg were developed in support of these studies (packing density = 202.7 mg/mL). This led to an increase in product density to ~69.8 mg/mL. A recent review by Weers et al. [56] provides considerations for spray drying suspension-based liquid feeds. This includes formulation and engineering solutions that enable maintenance of drug crystallinity irrespective of the physicochemical properties of the drug, including its aqueous solubility [56].

#### 4.2.3. Modifications of Packing Density

So far, the discussion has focused on modifications of particle density. Let us now turn attention to the packing of the particles in the powder bed. For gravitationally stable coarse particles ($d_{g}\;$> 100 μm), close packing within the powder bed is achieved with spherical particles [119,120,121]. Any decreases in sphericity increase bed porosity and reduce the packing density [119]. As particles become finer, increases in interparticle cohesive forces begin to negatively impact close packing of particles [119]. In this scenario it may be necessary to reduce cohesive forces via changes in particle morphology. These changes not only improve particle fluidization and dispersion, but they also enable close packing within the powder bed.

The impact of variations in surface corrugation on the packing density of fine cohesive powders comprising an antibody fragment (Fab) was studied by Son et al. [93,94].

The degree of surface corrugation is controlled through variations in the feedstock composition (e.g., solids loading, concentration of shell former) and by variations in the rate of drying. This can be described conceptually in the context of the Peclet number, a dimensionless number that describes the interplay of diffusion and evaporation in an atomized droplet during spray drying, viz [122,123,124,125,126]:

(3) $$Pe=\frac{\kappa }{D}=\frac{\mathrm{evaporation}\;\mathrm{rate}}{\mathrm{diffusion}\;\mathrm{rate}}\;$$

When an atomized droplet is dried slowly, the solutes within the liquid droplet have time to diffuse throughout the shrinking droplet. This leads to the formation of fine, higher density particles with a smooth particle morphology (i.e., no surface roughness). In contrast, if an atomized droplet is dried quickly relative to the diffusion rates of the solutes, the slow-diffusing solutes become enriched on the surfaces of the drying droplets, leading to low-density particles. Components with low solubility or high surface activity are concentrated at the interface of the receding droplet. The resulting particles are often hollow. Depending on the material properties of the shell, the particle may collapse upon drying to form a lower density corrugated morphology akin to a ‘wrinkled raisin’. By varying the Peclet number it is possible to control the degree of surface corrugation.

**Figure 3:**
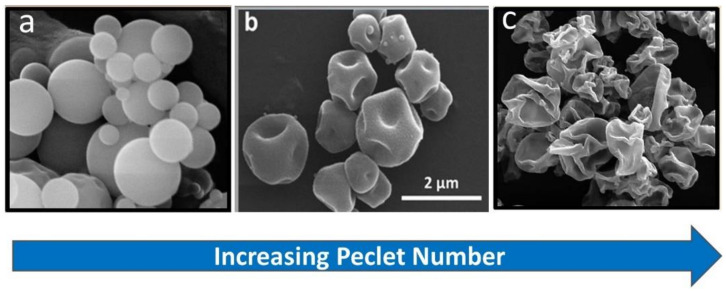
Scanning electron microscopy images of anti-TSLP Fab (CSJ117) particles spray dried with increasing Peclet numbers: (**a**) smooth spheres with 0% trileucine (Lot A7), (**b**) dimpled spheres with 2.5% trileucine (Lot A1), (**c**) corrugated spheres with 15% trileucine (Lot A13) [93].

The process conditions and micromeritic properties of three of the anti-TSLP Fab formulations studied by Son et al. [93,94] are summarized in Table 3. Scanning electron microscopy images of the particles are presented in Figure 3.

A modified compressibility index listed in Table 3 was defined to reflect parameters important for inhaled drug products comprising fine drug particles. Instead of using the poured bulk density and tapped density in the calculation, the inhaled compressibility index uses the tapped density and the puck density. The puck density is the bulk density measured under the compression used to form pucks during drum filling. While the standard (Carr’s) compressibility index and Hausner ratios provide information on the flowability of the bulk powder, these values are not reflective of the ability to fill these particles with high accuracy and precision on drum fillers, nor are they predictive of the resulting aerosol properties. The inhaled compressibility index provides a better metric to assess the impact of the degree of powder compression on filling and its resultant influence on aerosol performance [93,94]. Compressibility index values on the order of 10 indicate low degrees of powder compression on filling while values on the order of 40 indicate a high level of compression.

**Figure 4:**
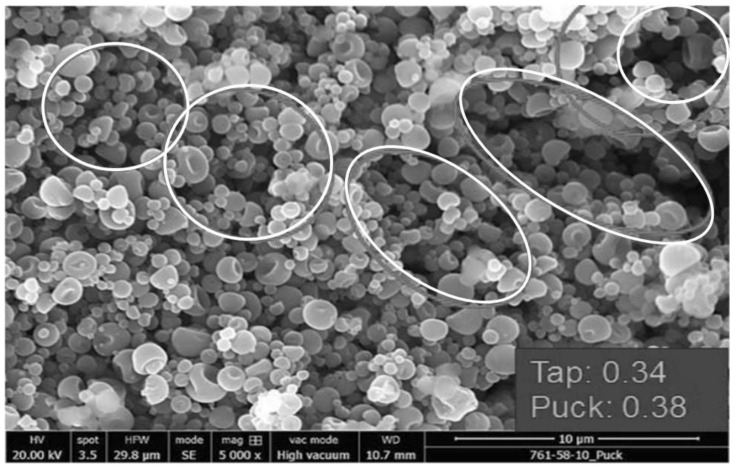
Scanning electron micrograph image of compressed spherical anti-TSLP Fab particles (Lot A7) showing large voids in powder bed [93].

In the absence of a shell-forming excipient such as trileucine, the spray-dried antibody forms smooth spheres (Figure 3a). Unfortunately, a high coordination number and packing density is not achievable with particles of this size; cohesive forces between the particles leads to particle bridging and large void spaces within the powder bed, even on compression to form a puck during drum filling (Figure 4). The puck density following compression of the spherical particles during filling was just 0.38 g/cm^3^.

The addition of 15% trileucine as a shell former and rapid drying conditions lead to significant increases in particle corrugation and specific surface area (SSA) for the core-shell powder formulation (Figure 3c). From the standpoint of packing density, the increased corrugation leads to a low particle density as the asperities on the particle surfaces prevent close packing. As a result, the puck density for the powder bed is just 0.28 g/cm^3^ (Table 3). This low puck density occurred despite the high compressibility index of this powder, i.e., the tapped density is significantly lower.

Introducing a small amount of corrugation within the particles (e.g., dimpling) by processing at a low Peclet number is sufficient to reduce the interparticle cohesive forces and improve packing within the powder bed (Figure 3b). For example, a powder with only 2.5% *w*/*w* trileucine manufactured using ‘slow’ drying conditions was able to pack more effectively, with a puck density of 0.64 g/cm^3^ (Table 3).

To be suitable for high dose powder delivery, a powder with an improved packing density must also empty and disperse effectively from the capsule during patient inspiration. The low compressibility index observed for the dimpled spheres suggests that the filling process may have an insignificant impact on powder dispersion [93,94]. For a 150 mg fill mass in a size 2 capsule (packing density = 405.4 mg/mL), the emitted dose of the dimpled particles was 83% after the first inhalation and 88% after the second inhalation [93,94]. The TLD was 0.69 mg/mg after the first inhalation, increasing to 0.83 mg/mg of the emitted dose after the second inhalation (2 L volume of air per actuation) [93,94]. This equates to a TLD of 110 mg of the dry powder and 55 mg of anti-TLSP Fab from a single size 2 capsule. The low Peclet Fab particles have a product density of 148.1 mg/mL (Table 2), which is about three-fold higher than is achieved by the small porous particle formulations presented in Table 1. A dramatic increase in product density occurs despite the formulation containing 50% excipients. The excipients include glass formers (trehalose, mannitol) and a buffer (histidine) to stabilize the antibody physically and chemically in an amorphous solid (glass), thereby enabling room temperature stability of the formulated drug product. A levofloxacin formulation spray dried at low Peclet numbers with a higher drug loading pushed the envelope even further, achieving a product density of 223.8 mg/mL (Table 2) [93].

The significant increases in packing density and product density achieved with these low Peclet number formulations enable delivery of high and ultrahigh TLD from a single capsule. While most studies to date have been conducted with DPIs utilizing size 3 and size 2 capsules, the design of two DPIs have been modified to incorporate larger sized capsules. Acorda Therapeutics modified the Turbospin device to increase the capsule size from size 2 to size 00 for the Inbrija drug product [25]. This markedly increased the receptacle volume from 0.37 to 0.95 mL. Similarly, the design of the RS01 DPI has been modified to incorporate size 2 and size 0 capsules from the original size 3 capsules [26,86]. The aerosol engines driving powder dispersion in these devices remain the same.

**Figure 5:**
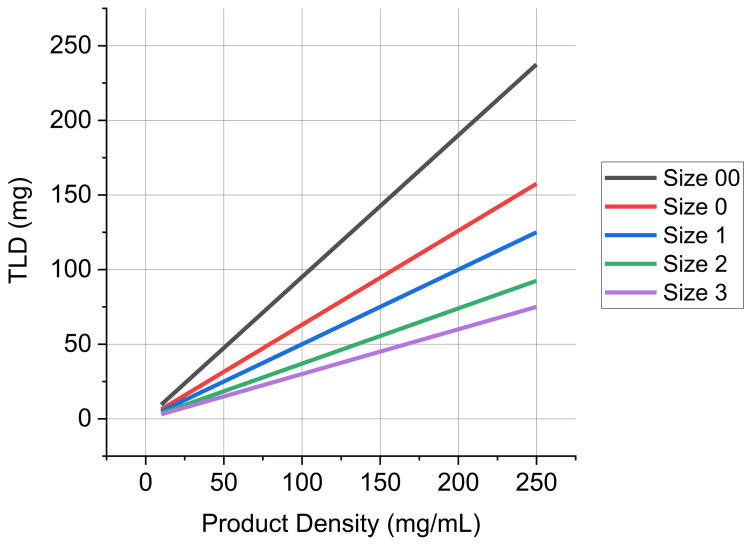
TLD as a function of the product density for various sized capsules.

Figure 5 displays the TLD as a function of the packing density for various sized capsules. For the products detailed in Table 1, the product densities are less than 50 mg/mL and they typically utilize size 2 and size 3 capsules. As such, it is clear from Figure 5 how the TLD/capsule is limited to about 20 mg. The impact of increasing product density and capsule size on the TLD/capsule is readily apparent. Ultrahigh TLD greater than 100 mg can be achieved at high product densities in size 0 and size 00 capsules for product densities greater than 160 and 110 mg/mL, respectively.

To put the product densities observed with low Peclet number formulations in perspective, the TLD for the ten administered capsules in Bronchitol is about 70 mg. In principle this could be accomplished by the inhalation of the contents of a single size 2 or size 0 capsule. Similarly, a product density of 200 mg/mL with tobramycin would allow for the administration of the current 70 mg TLD from a single size 2 capsule versus the four capsules in the current drug product.

Moving to a single larger-sized capsule to administer the full dose may not only reduce the daily treatment burden, administration time, and potential for capsule handling errors, it may also reduce the cost of goods (capsules, packaging), improve the shelf life (less moisture uptake in a larger dose), and reduce environmental impact (less packaging). Many patients, especially elderly patients, or those with neuropathy, may also find handling larger sized capsules to be easier.

Increasing the product density may also have important implications for blister-based multidose DPIs, potentially extending the range of TLD that can be achieved in this class of inhalers to 5–10 mg. This may enable less potent actives such as immunoglobulins, hormones, and kinase inhibitors to be delivered in a multi-dose DPI (MD-DPI) alone or in combination with potent asthma/COPD therapeutics.

### 4.3. Capsule Emptying

One concern with high dose delivery using passive DPIs is whether a patient’s inspiratory effort is sufficient to effectively fluidize and disperse the powder, and whether the inhaled volume of air is sufficient to empty powder from the capsule.

**Figure 6:**
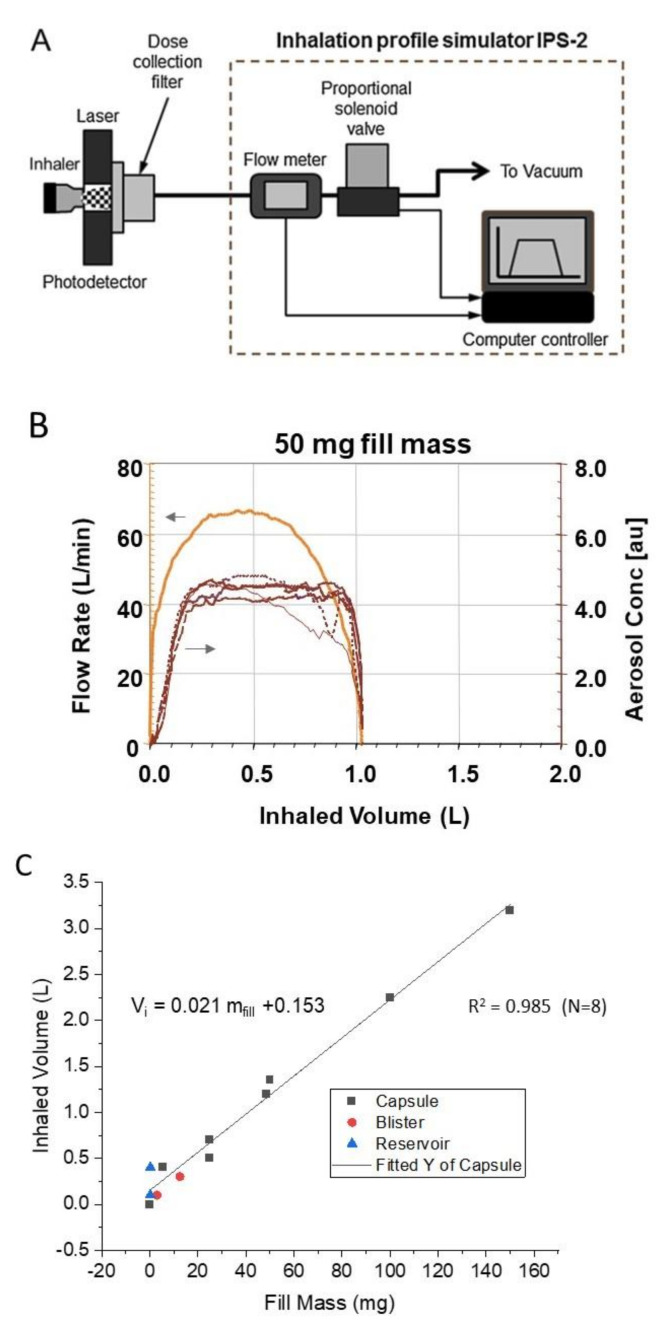
(**A**) Diagram of laser photometer system utilized to determine inhaled volume required to empty powder from a DPI [127,128]. The diagram was reproduced from [127] with permission from Elsevier, 2016; (**B**) Plot of a representative inspiratory flow profile and corresponding powder emptying profile for TOBI Podhaler using the laser photometer system; (**C**) Plot of the inhaled volume required to empty a fill mass for various inhaled products. DPIs included in the plot are: Asmanex^®^ Twisthaler^®^, Pulmicort^®^ Flexhaler^®^, Spiriva HandiHaler, Advair^®^ Diskus^®^, Onbrez Breezhaler, indacaterol Simoon™, vardenafil AOS^®^, Cipro T-326, TOBI Podhaler, CSJ117 T-326 [93,127,128]. It should be noted that the curve is dominated by capsule DPIs except at low fill masses.

The inhaled volume required to empty the powder contents from a capsule was determined by laser photometry [127,128]. The laser photometer generates a laser light sheet that intersects the flow path of the emitted aerosol immediately downstream of the inhaler mouthpiece (Figure 6A). The obscuration of the laser sheet caused by the emitted aerosol bolus is detected by a photodetector. The photodetector’s response is linear with obscuration and Beer’s Law is used to convert the response into a relative aerosol concentration. It is relative in the sense that it is not corrected for differences in scattering intensity that result from the presence of different sized particles in the laser light path over the period of powder emptying. The signal intensity is observed as a voltage pulse whose width corresponds to the duration of the aerosol emission process. Simulated inspiratory flow profiles are generated using a custom breath simulator. The breath simulator is equipped with a computer controlled proportional solenoid valve. When the system is connected to a vacuum source, the valve opening can be varied in a controlled manner to mimic a patient’s inspiratory flow profile.

Figure 6B illustrates the typical output for the laser photometer with a simulated inspiratory flow profile for a CF patient with the Podhaler device (*Vi* ~1.1 L and PIF ~66 L/min), along with the emptying profiles for four capsules containing 50 mg of tobramycin inhalation powder. For this inspiratory flow profile, the bulk of the powder is emptied with a single inhalation.

As the packing density increases within the same sized capsule, the inhaled volume needed to empty the powder increases in proportion to the mass of powder in the capsule (i.e., it is largely independent of the volume of powder) (Figure 6C).

For low dose asthma/COPD therapeutics, the inhaled volume required to empty the powder from reservoirs, blisters, and capsules is ≤0.7 L, with the upper end of the range being defined by capsule based DPIs with a 25 mg fill mass of lactose (Figure 6C). For the ~50 mg of powder in TOBI Podhaler, the required inhaled volume increases to about 1.1 to 1.2 L. The 150 mg fill mass for the anti-TSLP Fab in a size 2 capsule requires 3.3 L of inhaled volume to empty. For the 145 mg fill mass in Colobreathe, the predicted inhaled volume is ~3.2 L, while a 400 mg fill mass (e.g., Bronchitol) would require a minimum inhaled volume of 8.6 L to empty. The inhaled volume that subjects can achieve with portable dry powder inhalers depends on many factors, some of which are detailed in the examples below.

### 4.4. Inhaled Volumes of Patients

Azouz et al. [129] studied inspiratory flow profiles in 88 asthma and COPD patients. The mean inhaled volumes (*Vi*) were 1.10 L for pediatric asthma patients with an age of 8.8 ± 3.1 years, 1.62 L for adult asthma patients with a mean age of 48.7 ± 16.0 years, and 1.63 L for adult COPD patients with a mean age of 66.0 ± 9.6 years. Decreases in *Vi* were observed with increases in device resistance. Given that the *Vi* required for emptying asthma/COPD DPIs is ≤0.7 L, only a single inhalation would be needed, even for most pediatric patients. This is consistent with clinical practice.

Tiddens et al. [130] studied the inspiratory flow profiles of 96 patients with CF while inhaling through a test device containing a series of flow resistors. When inhaling against a resistance of 0.024 kPa^0.5^ L^−1^ min, 73% of children aged 6–10 years could achieve a *Vi* of 1.0 L. This decreased to 30% for a *Vi* of 1.5 L, and only 3% could reach a *Vi* of 2.0 L. For children with CF between the ages of 11–18 years, 92% could achieve a *Vi* of 1.0 L, decreasing to 62% of patients for a *Vi* of 1.5 L, and 33% for a *Vi* of 2.0 L. For adults older than age 18, 97% of patients could achieve a *Vi* of 1.0 L, 77% a *Vi* of 1.5 L, and 51% a *Vi* of 2.0 L. A small trend towards decreased *Vi* with increasing device resistance was observed. A marked decrease in *Vi* was noted with decreases in FEV_1_. Although most patients can achieve the ~1.2 L inhaled volume needed to empty a TOBI Podhaler capsule in a single inhalation, some cannot. To ensure that all patients receive their dose, the TOBI Podhaler instructions for use (IFU) call for two inhalations and subsequent inspection of the capsule to ensure that the dose is emptied. For Colobreathe (145 mg fill mass), 3–4 inhalations are needed by most patients to generate the 3.2 L of inhaled volume needed to empty the capsule. For the 120 mg fill mass of TobraPS, the *Vi* required to empty the powder from the size 0 capsule is about 2.7 L (i.e., 2–3 inhalations for most patients). Given that CF patients find that inhaling the contents of four capsules with two inhalations per capsule to be significantly more convenient and preferred relative to 15–20 min of jet nebulization, it is likely that 2–3 inhalations from a single capsule over a period of 1–2 min will not be a significant barrier to patient adherence. Nonetheless, factors that influence adherence with high dose powder delivery deserve more attention.

Haynes et al. [52] reported the inspiratory flow profiles of 38 patients with CF ranging in age from 6 to 71 years with the TOBI Podhaler device. Across all patients, inhaled volumes ranged from 0.6 to 3.1 L. For patients in the age groups 6–10, 11–18, and >18 years, the mean *Vi* values were 1.2, 1.6, and 2.1 L, respectively. Ten inhalation profiles that spanned the range of peak inspiratory flow (PIF) and *Vi* were selected for assessment of the TLD with the Alberta idealized throat model. As per the IFU, two inhalations were taken per capsule. The measured TLD was found to be independent of PIF and *Vi* over the range of simulated inhalation profiles, indicating that two inhalations are sufficient to achieve effective dose delivery to the lungs in most CF patients.

Luinstra et al. [89] assessed the inspiratory flow profiles of 13 adult patients with Parkinson’s disease with a test inhaler with resistance values varying from 0.037 to 0.061 kPa^0.5^ L^−1^ min. Inhaled volumes ranged from 1.2 to 3.5 L, with mean *Vi* increasing from about 1.8 to about 2.4 L as the device resistance decreased. It may be surprising to learn that in the Inbrija drug product, the large volume of low-density powder is emptied from the size 00 capsule in a single inhalation. The fill mass in Inbrija is 50 mg, suggesting that an inhaled volume of ~1.2 L should be sufficient to empty the capsule (Figure 6C). As described by Luinstra et al. [89], all the patients in their study achieved a *Vi* ≥ 1.2 L. Hence, the concerns expressed about using low density porous particles for ‘high dose delivery’, especially for moderate TLDs in the range from 1 to 20 mg, are unfounded [19]. Even the high 42 mg TLD requires only two inhalations from two capsules.

Sahay et al. [131] studied the inspiratory flow profiles of 35 adult patients with pulmonary arterial hypertension (PAH) using variants of the RS01 DPI that had resistance values between 0.017 and 0.051 kPa^0.5^ L^−1^ min. Mean *Vi* values increased from 1.7 to 1.9 L as the resistance of the device decreased. With the higher resistance DPI, the mean *Vi* decreased from 1.8 to 1.2 L when FEV_1_ values were decreased from >60% predicted to <50% predicted. Adult patients with PAH have lower inhaled volumes than adults with asthma, COPD, CF, or Parkinson’s disease, due in part to most PAH patients being female [131].

In summary, the inhaled volume that subjects achieve depends on many factors including their sex, age, the nature of their disease, the severity of their disease, and the inspiratory flow profile they achieve through the device. Most subjects inhale at only 40–80% of their maximum inspiratory pressure when using DPIs [132]. The effort that patients provide may also have an impact on their inhaled volume. The inhaled volume that subjects achieve is also dependent on the resistance of the DPI being utilized. For spray-dried formulations with minimal flow rate dependence, it may be advantageous to use a lower resistance DPI for high and ultrahigh dose delivery to minimize the number of inhalations required to empty the capsule. Achieving high dose delivery with a minimal number of inhalations is especially challenging in CF given the low *Vi* of many children.

## 5. Novel Devices for High Dose Delivery

At the top of the design considerations for a novel high dose DPI are the dose, treatment regimen, and intended patient population (sex, age, disease). The nature of the device may change significantly depending on whether the required TLD is moderate, high, or ultrahigh. The inhaled volume of women and children may be significantly lower than those of adult men, affecting the number of inhalations needed to empty a dose.

The device design must also consider the nature of the formulation. Will the powder be crystalline or amorphous? How will the dose be packaged? How will the dose be prepared for inhalation? How will the patient interact with the device? Additional questions to ponder are detailed below.

Is the intent to develop a single-use disposable device that can be emptied in a single inhalation or is the intent to develop a unit-dose device that accommodates a large receptacle with an ultrahigh powder dose that is emptied over multiple inhalations? For single-use disposable DPIs [133,134], the device is used once and then discarded. They are the preferred device when the drug product is administered just a few times (e.g., vaccines [135] or inhaled oxytocin for the prevention of post-partum hemorrhage [136]), when the dosing regimen calls for less frequent dosing (e.g., once weekly as in ABIP), or when the patient is immunocompromised and there is concern about the risk of infection when reusing the device. It has been suggested that single-use devices may also be preferred for amorphous powder formulations as accumulation of residual amorphous powder in the device can negatively impact device performance at elevated RH. For two marketed amorphous drug products (i.e., TOBI Podhaler and Exubera), this issue is effectively mitigated via modifications to the device and by shortening the device use life (or in the case of Exubera the transjector use life) to one week [34,137].

While single-use disposable DPIs may be suitable for the delivery of moderate doses in a single inhalation, questions remain regarding their utility in high dose chronic applications that may require many devices per day. To be successful in high dose delivery, the TLD/receptacle would need to be much greater than can be achieved with unit dose capsule DPIs with size 2 or 3 capsules. Otherwise, one is simply swapping a capsule for a device, which makes little sense.

For a device with a large volume receptacle, the critical question is, from a human-factors perspective, how many inhalations are reasonable before patients become nonadherent? Inhaling multiple times off the same receptacle may also lead to a more central deposition in the lungs as there will be no ‘chase’ air at the end of the inhalation to drive the powder peripherally. Is this acceptable for your drug product? Can the design help to minimize the inhaled volume needed to deliver the dose without negatively impacting powder dispersion and tolerability?

What will be the optimal device resistance be? As the device resistance increases, the inhaled volume that subjects achieve will tend to decrease. This can negatively impact the number of inhalations required to empty a large-volume receptacle. Alternatively, low resistance devices where subjects inhale at about 100 L/min may lead to increases in throat deposition for some formulations, thereby reducing aerosol performance and increasing variability.

With this as background, let us explore some of the new high dose device options. Comprehensive reviews of this topic are available [78].

### 5.1. Twincer^®^ and Cyclops^®^ DPIs

The Twincer and Cyclops are single-use disposable DPIs that use air classifiers to increase inertial forces acting on the powder to aid in powder dispersion [19,87,138]. The Cyclops DPI is about the size of a ~0.5 cm stack of credit cards. Powder formulations also include coarse lactose ‘sweeper’ crystals to increase the emitted dose by removing powder adhered to the walls of the device. The crystals are retained in the device and not inhaled by the subject. The intent with these devices is to maximize the TLD/mg powder by administering neat drug or formulations with minimal amounts of excipient and delivering these formulations with high efficiency to the lungs.

Both devices use a pre-loaded single-use cartridge with a peelable lidding foil. Delivery is simple and intuitive. After the foil strip is removed, the user inhales once, holds their breath for 5–10 s, and then discards the used device. Consistent with unit dose DPIs that employ a size 2 capsule, most subjects can empty and disperse up to ~50 mg fill mass from a standard single-dose cartridge (0.35 mL) in a single inhalation.

The inhaled volume required to empty approximately 50 mg of powder from a standard single-dose cartridge (0.35 mL) depends on the nature of the material and the flow rate/pressure drop attained by the inhalation maneuver. For the aminoglycoside amikacin, the complete dose is dispersed in about 1.0 L of inhaled volume at 6 and 4 kPa, and with about 1.5 L at a 2 kPa pressure drop [139]. These results are comparable to the ~1.2 L of inhaled volume required to empty a capsule of comparable volume in TOBI Podhaler.

For a 55 mg fill mass of isoniazid in the standard single dose cartridge (SDC), the drug emptied with a lower *Vi* of 0.23 to 0.33 L [140]. A larger (0.52 mL) SDC is also available. Sibum et al. [139] were able to achieve a fill mass of 150 mg of isoniazid by hand filling the entire SDC volume with drum-filled pucks (packing density = 288.5 mg/mL). The inhaled volume required to empty the powder was 0.38 to 0.43 L, significantly less than is reported to date for capsule inhalers.

Based on non-compendial laser diffraction testing in the absence of a throat, it was claimed that these devices achieve high fine particle fractions and low throat deposition in vivo [87,89]. Pharmacokinetic studies with tobramycin in bronchiectasis patients [88] and levodopa in Parkinson’s patients [90] suggest that lung delivery may be significantly less than suggested by these in vitro measurements. The dose-normalized systemic *C_max_* and *AUC* values observed with the Cyclops-based products are approximately two-fold lower than is observed with TOBI Podhaler and Inbrija (Section 4.1). These results suggest that lung delivery from the Cyclops is on the order of 25–35% of the nominal dose, which is consistent with the marketed Novolizer^®^ and Genuair^®^ DPIs that incorporate air classifier technologies [140,141]. Hence, while the Cyclops device is suitable for moderate dose delivery, it may have limited potential for chronic high dose delivery unless significant improvements in aerosol performance are achieved.

To put this in perspective, achieving equivalent delivery of levodopa as the Inbrija drug product (i.e., a TLD of 40 mg up to five times daily) with the clinical 30 mg Cyclops device would require approximately 900 devices/month ((6 devices/dose)(up to 5 doses/day)(30 days/month)) (Table 1, Table 2). For a 0.5 cm device thickness, this equates to a stack of devices 4.5 m high. Of course, the performance of the drug/device combination product could be optimized to decrease the number of required devices. However, even if the device delivered the entire 40 mg TLD in one inhalation from a single device, up to 150 devices would still be required per month. In our opinion, this type of chronic multidose/day high TLD product is not a practical application for the Cyclops technology. Additional work is needed to see if spray-dried formulations with high packing densities may enable significant increases in the dose loaded into a cartridge, in line with the isoniazid data pointed out above.

### 5.2. Orbital^®^ DPI

In contrast, the Orbital multi-breath DPI contains a large-volume cylindrical puck that has been demonstrated to encapsulate up to 400 mg of powder [27,142]. As such, it represents a step change in the mass of powder present in the receptacle compared to capsule based DPIs, providing a potential device solution for ultrahigh dose delivery.

To date, in vitro emptying studies have been conducted using an inhaled volume of 4 L. For mannitol-containing formulations, the emptying was independent of the fill mass, demonstrating a curious emptying profile where each inhalation decreases the mass of powder within the puck by about half, enabling emitted doses greater than 90% in about four ‘shots’.

In another study, Zhu et al. [142] demonstrated that emptying of tobramycin powder from the puck could be controlled by varying the diameter and number of holes in the puck. They selected a geometry that enabled a comparable dose of tobramycin to TOBI Podhaler to be delivered in four shots without the need to use multiple capsules (*Vi* = 16 L). The challenge with these emptying studies is that the 4 L inhaled volume is significantly larger than what CF patients can achieve (Section 4.4). Additional work is needed to better understand the hole size and number of holes required for optimal emptying with realistic inhaled volumes. The optimal emptying rate must also consider its impact on tolerability and powder dispersion. High emptying rates may lead to incomplete powder dispersion. This is true not only for the Orbital device, but for all large-volume receptacles, including the FB-DPI dosing sphere, and large volume capsules.

### 5.3. FB-DPI

The team at Virginia Commonwealth University have developed several novel DPIs including a fluidized bed high dose DPI (FB-DPI) [91]. In an early iteration of the device, drug product is loaded into a large-volume dosing sphere with a 10 mm inner diameter and four 0.5 mm holes. This equates to a receptacle volume of ~0.52 mL. The dosing sphere is placed within a bed of about sixty 4.76 mm PTFE mixing beads. When air is drawn through the device, the mixing beads fluidize, facilitating emptying and dispersion of powder from the dosing sphere. The powder then passes through a grid to provide secondary dispersion on the way to the patient. The FB-DPI is currently used in conjunction with VCU’s highly efficient excipient enhanced growth (EEG) formulation technologies [143]. In one study, Farkas et al. [91] loaded 100 mg of a ciprofloxacin EEG formulation containing mannitol, leucine, and poloxamer 188 into the dosing sphere (drug loading = ~0.30 mg/mg, packing density = 192.3 mg/mL) (Table 2). To assess aerosol performance, the flow rate was set at 60 L/min (4 kPa pressure drop), the inhaled volume at 4 L, and four inhalations were taken (*Vi* = 16 L). This resulted in an emitted dose of 71.4% and an FPF_<5 μm_ of 93.3% of the emitted dose. Given the flow rate, this is also a close approximation of FPF_S3-F_. This leads to aerosol performance of 0.67 mg/mg and a product density of 38.7 mg/mL. The TLD of ciprofloxacin was about 20 mg/dosing sphere. This is comparable to what was achieved with Ciprofloxacin DPI from a size 2 capsule with an inhaled volume of 1.3 L (Table 1).

## 6. Maximizing Safety and Tolerability at High Doses

Irritation or inflammation in the respiratory tract following pulmonary administration of inhaled therapeutics and excipients can cause treatment-emergent adverse events including bronchospasm, dyspnea, oropharyngeal pain, hoarseness, voice alteration, dysgeusia, wheezing, and cough. Adverse events are an important consideration in the development of inhaled therapeutics as they can result in poor adherence, discontinuation, and, ultimately, failure of treatment. Post-inhalation cough is the most reported adverse event for high dose delivery. Two recent reviews provide significant detail on post-inhalation cough with therapeutic aerosols [12,144].

Increases in the mass of drug and excipient deposited on epithelial lining fluid (ELF) can increase irritation and inflammation, irrespective of whether the drug is administered as a dry powder or a liquid aerosol. Nonetheless, there remains a bias that inhaled powders are inherently less tolerable than nebulized liquids.

Delivery of high doses of dry powder to the respiratory tract does not necessarily result in a direct irritant effect on the respiratory epithelium [12]. The nature of the material deposited in ELF matters. For example, lactose blends contain between about 12.5 and 25 mg of lactose. Most of the lactose is deposited near cough receptors in the upper respiratory tract. Yet this material does not cause airway irritation. Instead, drug or excipient materials that change osmolality, pH, or ion composition of ELF may activate release of leukotrienes and prostaglandins and cause local inflammation [12,144]. The magnitude of the effect may be potentiated by pulmonary disease and underlying inflammation in the lungs, especially for drugs with tussive potential.

Lung irritation, including cough, is impacted by the nature of the formulation. Cough may be elevated for salt forms of drugs with pK_a_ values less than 7.0 due to disproportionation of the salt in the ELF to form the free base and corresponding acid [12]. This can also occur with acidic drugs, disproportionating to the acid and corresponding base. The lower the pK_a_ of the acid used to make the salt, the greater the proton ion concentration on the epithelium, and the greater the irritant effect. The impact of salt disproportionation may be mitigated by using a neutral form of the drug, through the use of a cocrystal or by forming salts from acids with higher pK_a_ values [12]. This was demonstrated with inhaled bronchodilator, indacaterol, where a switch from the maleate salt (pK_a_ = 1.85) to the acetate salt (pK_a_ = 4.75) significantly reduced the incidence of post-inhalation cough [12,145,146].

**Figure 7:**
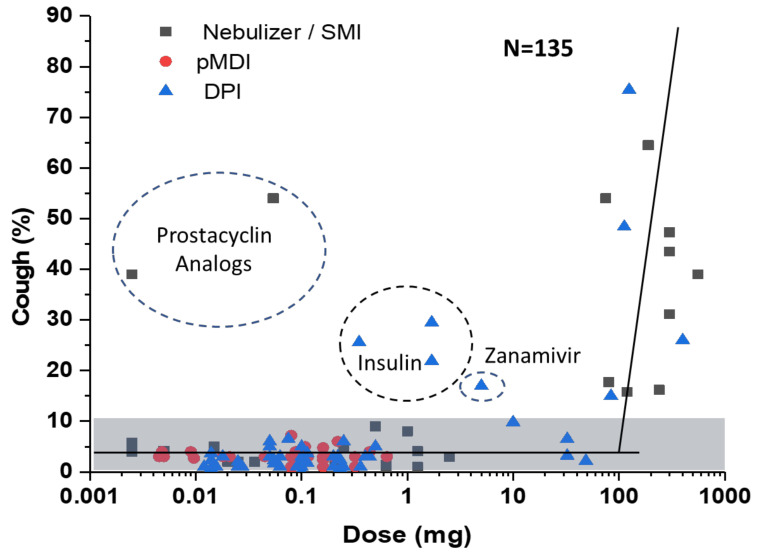
Impact of increases in nominal dose on the incidence of post-inhalation cough. The results are also presented with respect to the various delivery systems (square: nebulizers and soft mist inhalers (SMIs); circle: pressurized metered dose inhalers (pMDIs); triangle: dry powder inhalers (DPIs)). Reproduced with permission from [12]. Copyright Elsevier, 2020.

There is also a dose-dependent increase in airway irritation that is related to changes in osmolality via administration of ionizable species (e.g., salts) to ELF (Figure 7).

Post-inhalation cough increases markedly when the nominal dose of ionizable drugs exceeds about 10 mg [12]. For example, tobramycin contains five primary amine groups that can be protonated. When deposited in ELF, tobramycin sulfate produces a high osmolality in the vicinity of dissolving particles [12]. This is potentiated by the fact that cough receptors are enriched at bifurcations and other points where aerosols tend to accumulate [147]. In inhaled tobramycin, post-inhalation cough is generally mild to moderate in intensity and decreases with use. The intensity of the cough may be reduced by inhaling at lower flow rates. The cough threshold is lower in pediatrics than in adults.

In contrast to the high degree of cough observed with tobramycin sulfate, inhalation of the zwitterionic form of ciprofloxacin at neutral pH, at a comparable powder mass and with the same excipients, leads to minimal cough and airway irritation [12,54]. This is presumably due to the low osmolality of the poorly soluble neutral form of the drug in ELF [12,54]. Airway irritation can be mitigated by utilizing neutral forms of drugs, cocrystals instead of salts, and poorly soluble excipients [12]. For inhaled antibiotics, utilizing the poorly soluble neutral form of a molecule may have further utility due to its slow clearance from the lungs. This provides improved pulmonary targeting leading to improved pharmacokinetic/pharmacodynamic metrics (e.g., the ratio of *AUC*/*MIC*) and bacterial killing efficiency [54,148,149].

One concern with the use of poorly soluble drugs and excipients is that the presence of undissolved particulate matter in the lungs may result in adverse lung changes through a process of macrophage recruitment and stimulation with secondary lung damage and fibrosis [150]. However, there are examples from Table 1 where poorly soluble drug particles that are slowly cleared have no significant adverse effects in long-term nonclinical toxicology studies (e.g., ABIP, Ciprofloxacin DPI), and in long-term Phase 3 clinical studies [151,152]. Clearly, more studies are needed to better understand the link between slow particle clearance and adverse effects.

## 7. Conclusions

- Emptying of powder from capsules is highly dependent on the fill mass and less so on the fill volume. Most subjects can inhale approximately 40–50 mg of powder from a capsule in a single inhalation (requires ~1 L of inhaled volume).
- To date, the highest TLD/receptacle that can be emptied in a single inhalation for a marketed product is ~20 mg. This was achieved with Inbrija, a low density large porous particle formulation. This defines the upper limit for moderate TLDs (moderate TLD range is from 1 to 20 mg).
- For efficient high dose delivery (TLD = 20–100 mg), it is advantageous to maximize the $TLD/m_{powder}$. Maximizing dose delivery within this mass of powder requires achieving as high a TLD as possible with as little excipient as possible. The best spray-drying options to achieve this goal are: (1) lipid coated crystals prepared by spray drying suspension-based liquid feeds with ≤5% lipid excipient, and (2) core-shell particles prepared by spray drying a solution of drug and ≤5% of a shell forming excipient (e.g., trileucine) at low Peclet numbers.
- The mass of powder loaded into a given sized receptacle can be increased via increases in the packing density. This can be achieved to a high degree with the low Peclet core-shell particles noted above.
- For high dose delivery, target TLD values can be achieved from a single larger sized capsule with multiple inhalations needed to empty the capsule. The required inhaled volume is proportional to the mass of powder in the capsule. A 150 mg fill mass requires about 3.25 L of inhaled volume to empty (i.e., approximately three inhalations or less for most subjects). This enables a TLD of 80–100 mg from a single size 2 capsule.
- Ultrahigh doses (TLD > 100 mg) require large receptacles (e.g., size 0 or size 00 capsules) or novel DPIs (e.g., the large volume cylindrical puck in the Orbital DPI or the dosing sphere in the FB-DPI).
- It is critical to better understand how the size and number of holes in larger sized receptacles impact powder emptying. Further, it is critical that these emptying studies be conducted with realistic patient breathing profiles, as opposed to the standard 4 L inhaled volumes typically used in aerosol testing.
- Evidence established to date suggests that high doses of neat drug particles (sans excipient) have significantly decreased aerosol performance and poor dose consistency. The addition of small amounts of shell forming excipient is necessary to improve aerosol performance, packing density, dose consistency, and, ultimately, product density.
- The inhalation of large doses of powder does not lead to increases in local irritation in the lungs. It depends on the nature of the powder inhaled.
- While significant progress has been made in increasing the dose of powder that can be delivered from a portable DPI, we have limited understanding of the practical limits of high dose powder delivery both from a tolerability perspective, but also from a patient adherence perspective. How many inhalations are practical and at what point is a liquid nebulizer or possibly a powder nebulizer a better solution?
- Overall, ultrahigh doses of drug (>100 mg) can be deposited in the lungs with a portable DPI in three to four inhalations when using a device with a receptacle volume greater than 0.6 mL and spray-dried porous particles with a high packing density greater than 150 mg/mL.

## Figures and Tables

**Table 1:** Product density metrics for various late-stage and marketed products.

Drug Product	Packing Density (mg/mL)	Drug Loading (mg/mg)	Aerosol Performance (mg/mg)	Product Density (mg/mL)
Spiriva^®^ HandiHaler^®^	18.3	0.003	0.20	0.012
Onbrez^®^ Breezhaler^®^	83.3	0.006	0.34	0.17
Intal^®^ Spinhaler^®^	54.1	0.91	0.11	5.4
Relenza^®^	125.0	0.20	0.23	5.8
Colobreathe^®^	391.9	0.44	0.12	20.7
TOBI^®^ Podhaler™	131.1	0.58	0.63	47.1
Ciprofloxacin DPI	135.1	0.65	0.53	46.5
ABIP	27.0	0.50	0.70	9.5
Capreomycin DPI	100.0	0.69	0.30	20.7
Aerovanc^®^	100.0	0.83	0.50	41.5
Bronchitol^®^	133.3	1.0	0.18	23.3
Inbrija^®^	52.6	0.84	0.50	22.1

**Table 2:** Product densities from selected research studies.

Drug Product	Packing Density (mg/mL)	Drug Loading (mg/mg)	Aerosol Performance (mg/mg)	Product Density (mg/mL)
Clofazimine RS01 [83]	66.7	1.0	0.45	30.0
Tobra Form I RS00 [84]	83.3	0.95	0.53	41.9
Tobra Form 2 RS00 [84]	83.3	1.0	0.34	28.4
TobraPS RS01 [85,86]	176.5	0.91	0.65	104.4
Tobra Cyclops [87,88]	85.7	0.95	0.34	27.7
Levodopa Cyclops [89,90]	85.7	0.98	0.24	20.2
Ciprofloxacin FB-DPI [91]	192.3	0.30	0.67	38.7
Levofloxacin iSPERSE [92]	133.3	0.51	0.40	48.0
TSLP Fab T-326 [93,94]	405.4	0.50	0.73	148.1
Levofloxacin T-326 [93]	405.4	0.80	0.69	223.8
FDC R/K RS00 [95]	66.7	1.0	0.61	40.7

**Table 3:** Process conditions and micromeritic properties of three formulations comprising 50% *w*/*w* of an anti-human thymic stromal lymphopoietin (TSLP) antibody fragment (CSJ117) [93,94]. Adapted with permission from Virginia Commonwealth University and Respiratory Drug Delivery, 2020.

Anti-TSLP Fab (CSJ117)	Lot A7	Lot A1	Lot A13
Morphology	Smooth spheres	Dimpled spheres	Corrugated particles
Trileucine (% *w*/*w*)	0	2.5	15
Solids Loading	1.0	1.0	1.0
Drying conditions ^a^	Fast	Slow	Fast
×50 (μm) ^b^	1.19	1.43	1.36
×90 (μm) ^b^	1.87	2.39	2.94
SSA (m^2^/g) ^b^	6.35	6.01	14.90
Bulk density (g/cm^3^)	0.21	0.29	0.10
Tapped density (g/cm^3^)	0.34	0.57	0.15
Puck density (g/cm^3^)	0.38	0.64	0.28
Compressibility (inh)	10.5	10.9	46.4

^a^ Slow drying conditions: feed rate = 2.5 mL/min; outlet temperature = 55 °C; airflow rate = 300 L/min. Fast drying conditions: feed rate = 5.0 mL/min; outlet temperature = 70 °C; airflow rate = 600 L/min. ^b^ ×50: 50% of the particles in the volume distribution have a primary particle size less than the ×50; ×90: 90% of the particles in the volume distribution have a primary particle size less than the ×90; SSA: specific surface area.
